# Breakthroughs in soybean transformation

**DOI:** 10.1093/plphys/kiaf678

**Published:** 2025-12-26

**Authors:** Neeta Lohani

**Affiliations:** Assistant Features Editor, Plant Physiology, American Society of Plant Biologists; Department of Biotechnology, Thapar Institute for Engineering and Technology, Patiala, Punjab 147004, India

Soybean (*Glycine max* (L.) Merrill) is a globally important legume and oilseed crop, with seeds containing approximately 40% protein and 20% oil on a dry weight basis (Singer et al. 2023). As a legume, soybean also contributes to sustainable agriculture through symbiotic nitrogen fixation, reducing dependence on synthetic fertilizers. Advances in genomics have identified several candidate genes for agronomically important traits, yet a fundamental disconnect exists between gene discovery and functional validation. Conventional tissue culture–based transformation protocols typically achieve frequencies below 5%, require 16 to 20 weeks of labor-intensive culture, and work effectively in only a handful of genotypes (Xu et al. 2022; Zhong et al. 2024). This transformation bottleneck has become the rate-limiting step for developing genetically engineered varieties with desirable traits (Freitas-Alves et al. 2025). The challenge lies not in DNA delivery, which *Agrobacterium*-mediated methods accomplish efficiently, but in regeneration, which is the process of inducing transformed cells to form shoots and fertile plants.

In a recent issue of *Plant Physiology*, 2 studies reported significant advances in soybean transformation. Alok et al. (2025) demonstrated that developmental regulators WUSCHEL2 (WUS2) and isopentenyltransferase (IPT) can be coexpressed to dramatically enhance transformation efficiency while eliminating the need for exogenous hormones, completing the entire process in just 9 to 11 weeks (60 to 80 days). Li et al. (2025) described ROTIS (RUBY-assisted One-shot Tissue-culture-free In-planta Soybean-transformation), a complementary system that bypasses sterile tissue culture entirely by using wounded apical meristems and *Agrobacterium* infiltration under nonsterile conditions, achieving transformation across 21 diverse genotypes in 40 to 80 days.

Alok et al. (2025) systematically evaluated multiple developmental regulator (DR) combinations for their ability to promote de novo shoot formation directly from germinating soybean embryos, bypassing the lengthy callus induction phase, which is typical of conventional transformation protocols. Their results revealed striking performance differences between the different DR combinations. WUS2 and IPT alone yielded only 3.3% and 6.4% transformation efficiency, while the GRF4-GIF chimera, which has succeeded in other systems under hormone-supplemented conditions, failed without exogenous hormones in the media. In contrast, WUS2/IPT coexpression achieved 14.6% to 22.3% transformation efficiency in Williams 82 and Bert varieties (Fig. 1), suggesting mechanistic synergy rather than simple additive effects. The streamlined workflow takes just 9 to 11 weeks from embryo isolation to T1 seed harvest, representing a 25% to 45% reduction compared to the 16 to 20 weeks required by conventional methods. Additionally, this approach eliminates the need for selection agents, as transgenic shoots can be visually identified by the distinctive red-purple pigmentation produced by the RUBY reporter. Further demonstrating the efficiency and utility of this system for genome editing, the authors combined it with CRISPR-Cas9 to target the *P34* allergen gene, generating heritable mutations at 20% efficiency. These results demonstrate that the DR-enabled approach effectively delivers CRISPR components and produces stable edits at frequencies comparable to conventional methods.

**Figure 1.**
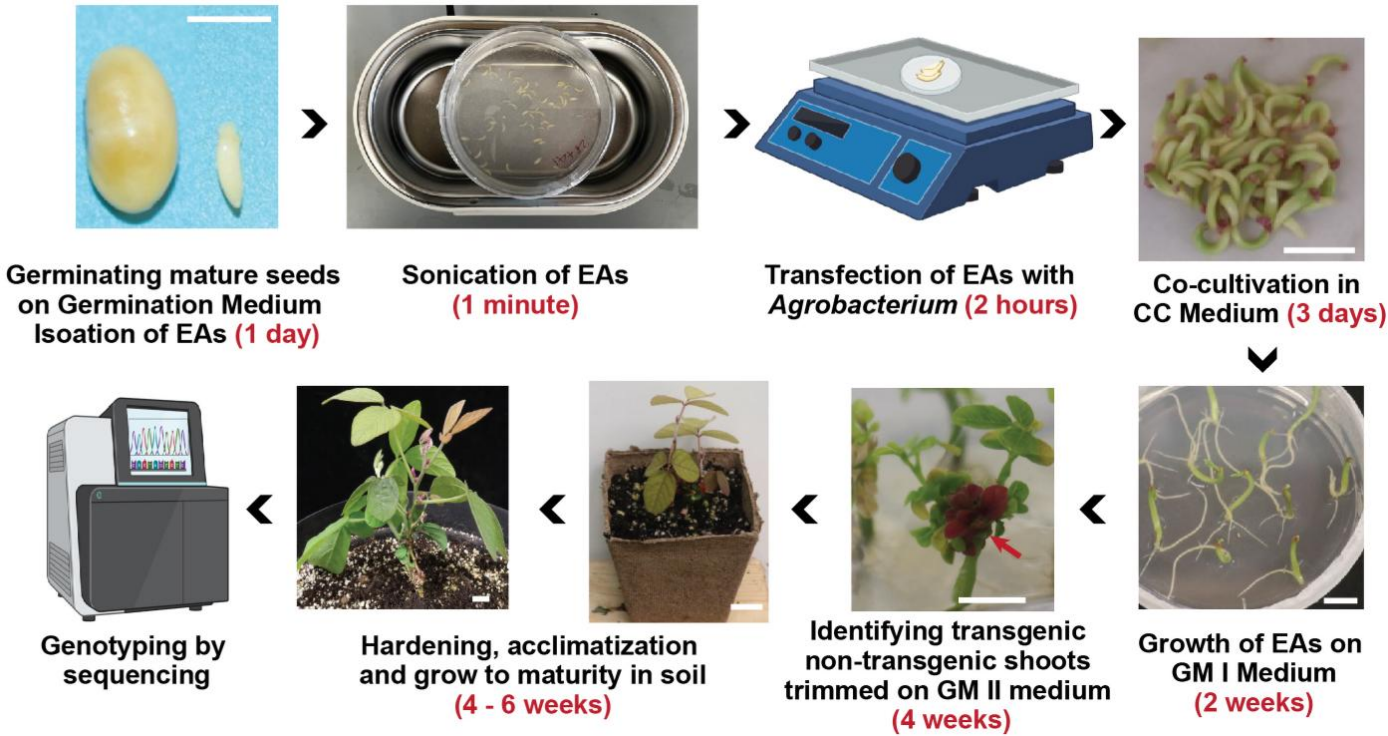
WUS2/IPT developmental regulator-mediated soybean transformation. The method uses germinating embryos as explants, transformed with *Agrobacterium* carrying WUS2, IPT, CRISPR-Cas9, and the RUBY visual marker. Explants are cultured on hormone-free media, and transgenic shoots are identified by distinctive red-purple coloration. The entire process from embryo isolation to T1 seed harvest takes 9 to 11 weeks. Figure adapted from Alok et al. (2025).

Next, to understand the molecular basis of this synergy, the authors performed temporal transcriptomic analysis at 3 and 6 days after transformation. Their findings reveal a carefully orchestrated developmental program. At 3 days, WUS2/IPT simultaneously suppressed stress-response genes triggered by *Agrobacterium* infection while activating cell cycle and hormone signaling pathways. This dual action proves crucial because defense responses typically hinder transformation efficiency. By 6 days after transformation, hormone-responsive gene analysis showed a shift from stress hormones (jasmonic acid, abscisic acid, ethylene) to growth-promoting hormones (auxin, cytokinin). Strong upregulation of cytokinin signaling, meristem identity genes, and auxin pathways provided molecular signatures consistent with de novo shoot formation. Notably, WUS2 alone clustered with empty vector controls at 6 days rather than with IPT, explaining its poor solo performance and confirming that WUS2 requires hormone support to drive regeneration effectively.

Li et al. (2025) developed ROTIS, a system that bypasses sterile tissue culture requirements. Seeds are germinated in perlite, and once the cotyledons expand, the apical meristem is excised to create a wound site. Explants are vacuum infiltrated with *Agrobacterium* suspension, and *Agrobacterium*-soaked cotton balls are placed on the wounded meristem to maintain contact during co-cultivation. Adventitious shoot formation is induced by replacing these with shoot induction medium-soaked cotton balls. The entire process occurs under nonsterile conditions. The RUBY reporter enables visual identification of transgenic shoots within 3 to 5 days, while kanamycin leaf painting provides additional selection. From seed germination to T0 seed harvest, the pipeline requires only 40 to 80 days (Fig. 2). The system achieved consistent transformation across 21 diverse soybean varieties, including elite cultivars from northeast China, with RUBY-positive shoot frequencies up to 35.7%.

**Figure 2.**
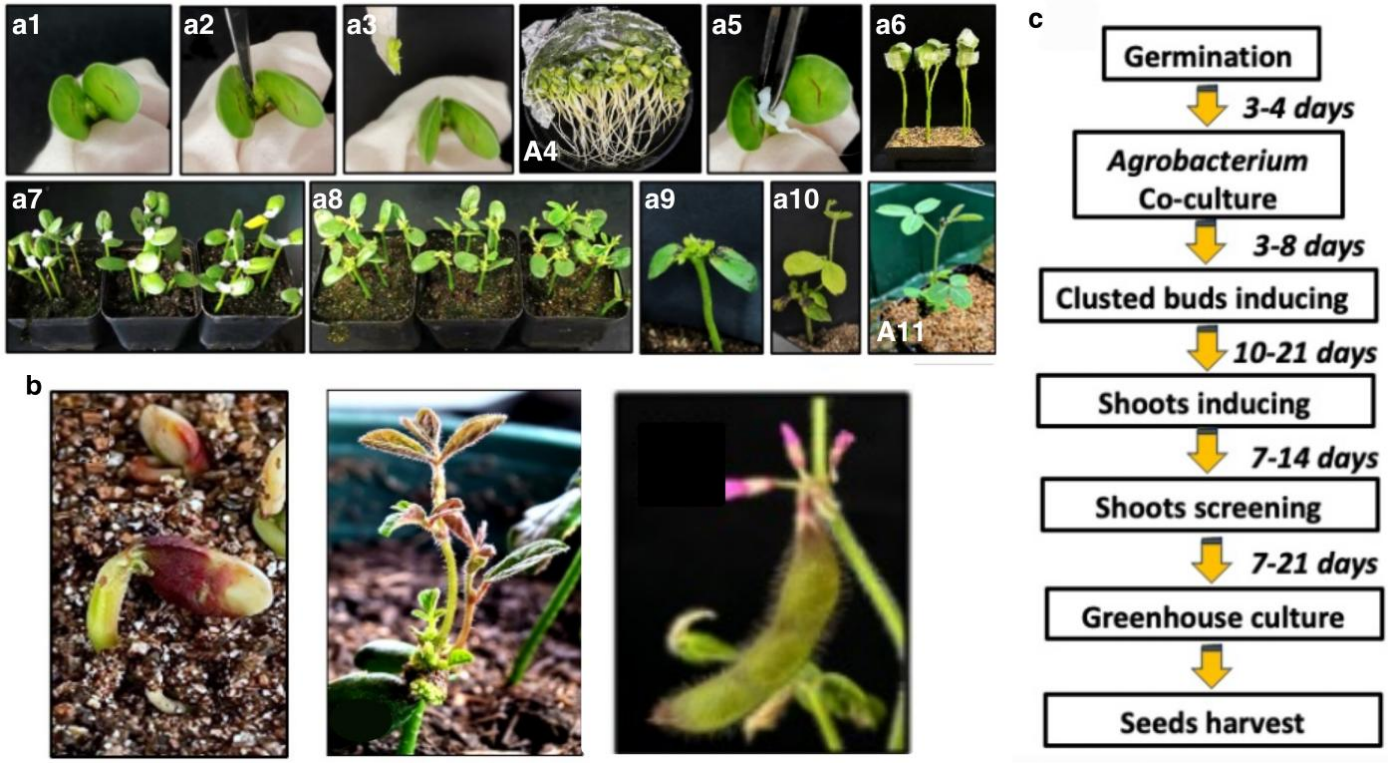
ROTIS tissue culture–free soybean transformation system. **a)** Workflow showing germinated seedling explants (A1), apical meristem wounding (A2–A3), vacuum infiltration with *Agrobacterium* (A4), seedling recovery (A5–A6), shoot cluster induction and growth (A7–A10), and RUBY-positive transgenic plant (A11). **b)** RUBY reporter phenotypes: early red pigmentation at the shoot base, mature plant with red-pigmented leaves, and pink flower in transgenic line. **c)** Timeline of the ROTIS pipeline from germination to seed harvest. Figure adapted from Li et al. (2025).

Both studies employ the RUBY reporter to visually identify transgenic events through distinctive red pigmentation, enabling nondestructive screening that streamlines the selection process. While sharing this visual marker strategy, the 2 approaches differ substantially in their technical requirements and demonstrated capabilities. The WUS2/IPT method operates within a sterile tissue culture framework and harnesses morphogenic regulators to drive regeneration, an approach that proved compatible with CRISPR-Cas9 delivery and achieved 20% heritable mutation efficiency when targeting the *P34* allergen gene. ROTIS takes a fundamentally different approach by eliminating the requirement for both tissue culture and morphogenic regulators, instead transforming germinated seedlings under nonsterile conditions using *Agrobacterium*-soaked cotton balls applied to wounded apical meristems. This simplified workflow achieved consistent transformation across 21 diverse genotypes, effectively addressing the genotype dependency that has historically limited soybean transformation. Its capacity for genome editing, however, has not been evaluated. Furthermore, both methods present opportunities for further refinement, particularly regarding the effects of RUBY expression on plant development. Alok et al. (2025) reported that strong constitutive RUBY expression reduced plant vigor, with transgenic tissues noticeably smaller than their nontransgenic siblings; this effect was not observed in the ROTIS study, possibly reflecting differences in expression levels, promoter strength, or genetic backgrounds. Additionally, variable transgene inheritance patterns in both methods suggest the occurrence of chimerism, a challenge that future work might address through inducible promoter systems or recombinase-mediated transgene excision.

Inefficient transformation has long restricted genome editing to a few model cultivars, requiring lengthy backcrossing to transfer beneficial alleles into elite germplasm. These advances in soybean transformation methods have practical implications for soybean improvement. Both methods now enable direct transformation of commercial varieties, shortening the timeline from gene discovery to varietal improvement. This is particularly valuable for trait stacking, which is essential for developing climate-resilient varieties with combined stress tolerance, enhanced nutrition, and improved agronomic performance. The tissue culture–free ROTIS system also makes soybean transformation accessible to laboratories without specialized infrastructure.

Several important questions remain for future investigation, including whether ROTIS can achieve efficient genome editing comparable to the WUS2/IPT system, whether alternative visual markers might reduce the metabolic burden associated with constitutive RUBY expression, and whether the WUS2/IPT method can be successfully extended beyond Williams 82 and Bert to a broader panel of globally important cultivars. These studies build on pioneering work with morphogenic regulators in maize (Lowe et al. 2016) and GRF-GIF innovations in wheat (Debernardi et al. 2020), demonstrating that creative reimagining of transformation strategies can overcome barriers that incremental protocol optimization alone cannot address. Together, these methods will help accelerate the development of soybean varieties better suited to meet future agricultural challenges.

## Related Articles

Li Q et al. (2025) A simplified *Agrobacterium tumefaciens*–mediated transformation protocol accelerates plant molecular breeding. Plant Physiology 197(1): kiae585

Kuwabara C et al. (2024) A DNA-free and genotype-independent CRISPR/Cas9 system in soybean. Plant Physiology 196(4): 2320–2329
